# Crystalline versus non-crystalline light-chain proximal tubulopathy: a case series

**DOI:** 10.3389/fmed.2026.1963349

**Published:** 2026-10-14

**Authors:** Zhi-Rong Zhang, Li-Mei Wang, Xiao-Le Meng, Ting Zhu, Zhi-Gang Zhang, Zhong-Hua Zhao, Wei-Xia Wang, Si-Nian Huang

**Affiliations:** 1 Department of Pathology, QingPu Branch, Zhongshan Hospital Affiliated to Fudan University, Shanghai, China; 2 Department of Pathology, Zhongshan Hospital (Xiamen Branch), Fudan University; Clinical Research Center for Precision Medicine of Abdominal Tumor of Fujian Province, Xiamen, China; 3 Department of Pathology, School of Basic Medical Sciences, Fudan University, Shanghai, China

**Keywords:** autologous stem cell transplantation, crystalline LCPT, IgD multiple myeloma, light-chain proximal tubulopathy, non-crystalline LCPT

## Abstract

Light-chain proximal tubulopathy (LCPT) represents a rare paraprotein—associated kidney disorder resulting from the intracellular accumulation of monoclonal free light chains within proximal tubular epithelial cells, and manifests as either crystalline or non-crystalline inclusions. Crystalline LCPT is most frequently observed in IgG-*κ* multiple myeloma. Conversely, IgD-*λ* multiple myeloma typically presents as the non-crystalline variant. The histopathological findings, clinical correlations, and therapeutic outcomes differ substantially between these two entities. This study comparatively analyzed two LCPT cases and summarized the clinicopathological characteristics, diagnostic strategies and treatment outcomes of different LCPT subtypes. The 55-year-old male patient with IgG-*κ* multiple myeloma presented with renal insufficiency, and renal biopsy confirmed crystalline LCPT. Typical crystalline structures in proximal tubular epithelial cells and *κ* light chain deposition were verified by light, electron and immunoelectron microscopy. The patient achieved favorable hematologic and renal prognosis after bortezomib-based chemotherapy combined with autologous hematopoietic stem-cell transplantation (ASCT). The 64-year-old male patient with IgD-*λ* multiple myeloma suffered from progressive renal failure. Biopsy revealed non-crystalline LCPT characterized by non-crystalline lysosomal lesions with concurrent light-chain cast nephropathy, characterized by non-crystalline lysosomal lesions and *λ* light chain accumulation. He received multiple cycles of bortezomib-based chemotherapy without ASCT and finally died of multiple organ failure. Literature review indicates that LCPT morphological phenotypes are closely correlated with light chain subtypes. IgD-*λ*-related non-crystalline LCPT is rarer, more aggressive, and prone to severe renal impairment. In conclusion, for patients with unexplained proximal tubular dysfunction and monoclonal gammopathy, early precise diagnosis relying on ultrastructural examination and light chain immunohistochemical detection is critical. Bortezomib combined with ASCT is the optimal treatment for LCPT to achieve hematologic remission and preserve renal function, while close attention is required for high-risk IgD-*λ* non-crystalline LCPT.

## Introduction

Light-chain proximal tubulopathy (LCPT) represents a relatively uncommon renal disease that typically arises from monoclonal gammopathies in patients with multiple myeloma or monoclonal gammopathy of renal significance (MGRS). First described in 1975 (1), this disorder results from the intracellular accumulation of monoclonal free light chains (FLCs) within the cytoplasm of proximal tubular epithelial cells, leading to tubular dysfunction, Fanconi syndrome, and progressive chronic kidney disease (2, 3). Fanconi syndrome is a proximal tubular dysfunction characterized by impaired reabsorption of phosphate, bicarbonate, potassium, glucose, amino acids, and low-molecular-weight proteins, resulting in glycosuria, aminoaciduria, phosphaturia, proximal renal tubular acidosis, hypokalemia, and hypophosphatemia. According to the presence or absence of crystalline structures within tubular cells on electron microscopy, LCPT is classified into two subtypes: crystalline and non-crystalline (4).

Clinically, LCPT is most commonly associated with *κ* light-chain deposition, with the majority of reported cases occurring in patients with monoclonal IgG-type multiple myeloma (5). A minority of cases involve IgA, IgM, or IgD myelomas. Immunoglobulin D multiple myeloma (IgD-MM) is an aggressive subtype, comprising less than 2% of all multiple myeloma cases (6). LCPT arising in the setting of IgD-*λ* MM is exceedingly rare, with both crystalline (7) and non-crystalline (8) forms having been documented.

Therefore, the pathogenic mechanisms and clinicopathological manifestations of crystalline versus non-crystalline proximal tubulopathy remain distinct. In this article, we report two cases of LCPT: one of IgG-*κ* type with crystalline inclusions, and the other of IgD-*λ* type without crystalline formation and with concurrent cast nephropathy. In addition, we review the pertinent literature to further explore the histopathological features of these two subtypes, the diagnostic approach to LCPT, and the implications of differential therapeutic strategies.

## Case presentation

### Case 1: crystalline LCPT (igG-*κ* restricted type)

A 55-year-old male was admitted on February 17, 2019, with a two-year history of abnormal urinalysis. Laboratory findings are summarized in Table 1. Relevant findings included hemoglobin 131 g/L, platelet count 191 × 10⁹/L, serum IgG 15.80 g/L, serum free *κ* light chains were markedly elevated (427.5 mg/L), with a *κ*/*λ* ratio of 50.47. Serum creatinine was 114 μmol/L with an eGFR of 61.6 mL/min/1.73 m^2^. Twenty-four-hour urinary protein was 1,259 mg. Serum calcium was 2.38 mmol/L, phosphate 1.36 mmol/L, potassium 3.7 mmol/L, bicarbonate 22.7 mmol/L, and uric acid 300 μmol/L. LDH was 143 U/L.

**Table 1:** Baseline clinical, laboratory, and pathological characteristics of the two cases.

Parameter	Case 1 (Crystalline LCPT)	Case 2 (Non-crystalline LCPT with Cast Nephropathy)
Age/Sex	55 years/Male	64 years/Male
Admission date	2019-02-17	2019-08-15
DS stage	IIIB	III
ISS stage	II	III
R-ISS stage	III	III
Bone marrow plasma cells	15% (biopsy); 2% (flow)	77% (smear); 36.7% (flow)
FISH/cytogenetics	TP53 deletion (+), 1q21 (+), 13q14 (+), IgH (−)	1q21+; TP53 not available; karyotype 46, XY (20)
Hemoglobin (g/L)	131	104
Platelet count (×10⁹/L)	191	130
Serum IgG (g/L)	15.80	4.14 (decreased)
Serum free *κ* (mg/L)	427.5	9.54
Serum free *λ* (mg/L)	8.47	31,550
κ/λ ratio	50.47	0.0003
Serum creatinine(*μ*mol/L)	114	1,021
eGFR (mL/min/1.73 m^2^)	61.6	4.1
24-h urinary protein (mg)	1,259	2,060
Serum albumin (g/L)	43	48
Serum calcium (mmol/L)	2.38	2.47
Serum phosphate (mmol/L)	1.36	1.73
Serum potassium (mmol/L)	3.7	3.8
Serum bicarbonate (mmol/L)	22.7	18.7
Serum uric acid (μmol/L)	300	811
LDH (U/L)	143	297 (peak 1,961)^a^
Urine glucose	Positive (++)	Negative
Urinary pH	7.5	7.5
Urinary *β*2-microglobulin (mg/L)	30.7	10.2
Urinary amino acids	Not performed	Not performed
Diagnosis	Crystalline LCPT (IgG-κ type)	Non-crystalline LCPT with concurrent light-chain cast nephropathy (IgD-λ type)
Intraluminal casts	Absent	Present (light microscopy)
Mass spectrometry	Not performed	Not performed

DS, Durie–Salmon; eGFR, estimated glomerular filtration rate; FISH, fluorescence *in situ* hybridization; Ig, immunoglobulin; ISS, International Staging System; LDH, lactate dehydrogenase; LCPT, light-chain proximal tubulopathy; R-ISS, revised International Staging System.

a Serum LDH in Case 2 was 297 U/L at admission and peaked at 1,961 U/L on August 30, 2019.

Immunofixation electrophoresis revealed a monoclonal IgG-*κ* peak. Positron emission tomography–computed tomography (PET-CT) showed multiple lytic bone lesions. Bone marrow biopsy showed 15% plasma cells, and flow cytometry identified 2% monoclonal plasma cells with a CD38 ^+^ CD138 ^+^ CD56^partial^ ^+^ CD19^−^CD20^−^CD117^+^ phenotype and intracellular *κ* light-chain restriction. FISH analysis revealed high-risk cytogenetic abnormalities: TP53 deletion, 1q21 amplification, and 13q14 deletion; IgH rearrangement was negative. The patient was diagnosed with multiple myeloma, DS stage IIIB, ISS stage II, R-ISS stage III.

Renal Biopsy Findings: Light microscopy revealed 19 glomeruli, of which 4 exhibited global sclerosis. The remaining glomeruli showed mild mesangial cell proliferation with a slight increase in mesangial matrix. Some proximal tubular epithelial cells appeared swollen, with granular degeneration evident in their cytoplasm (Figure 1a). PASM staining demonstrated diffuse, deep-brown, polymorphic granules within the cytoplasm of proximal tubular cells (Figures 1b,c). Masson's trichrome staining highlighted red cytoplasmic granules (Figure 1d). Congo red staining was negative. Immunohistochemistry showed strong cytoplasmic positivity for *κ* light chains in proximal tubule cells (Figure 1e), and immunofluorescence on protease-digested paraffin sections confirmed *κ* light-chain restriction (Figure 1f). Electron microscopy disclosed clusters of pleomorphic rhomboid or polygonal crystals distributed throughout the cytoplasm of proximal tubular epithelial cells. Immunoelectron microscopy with colloidal gold labeling confirmed these crystals to be specifically labeled by anti-*κ* antibodies (Figures 1g–i). No intraluminal casts were identified.

**Figure 1:**
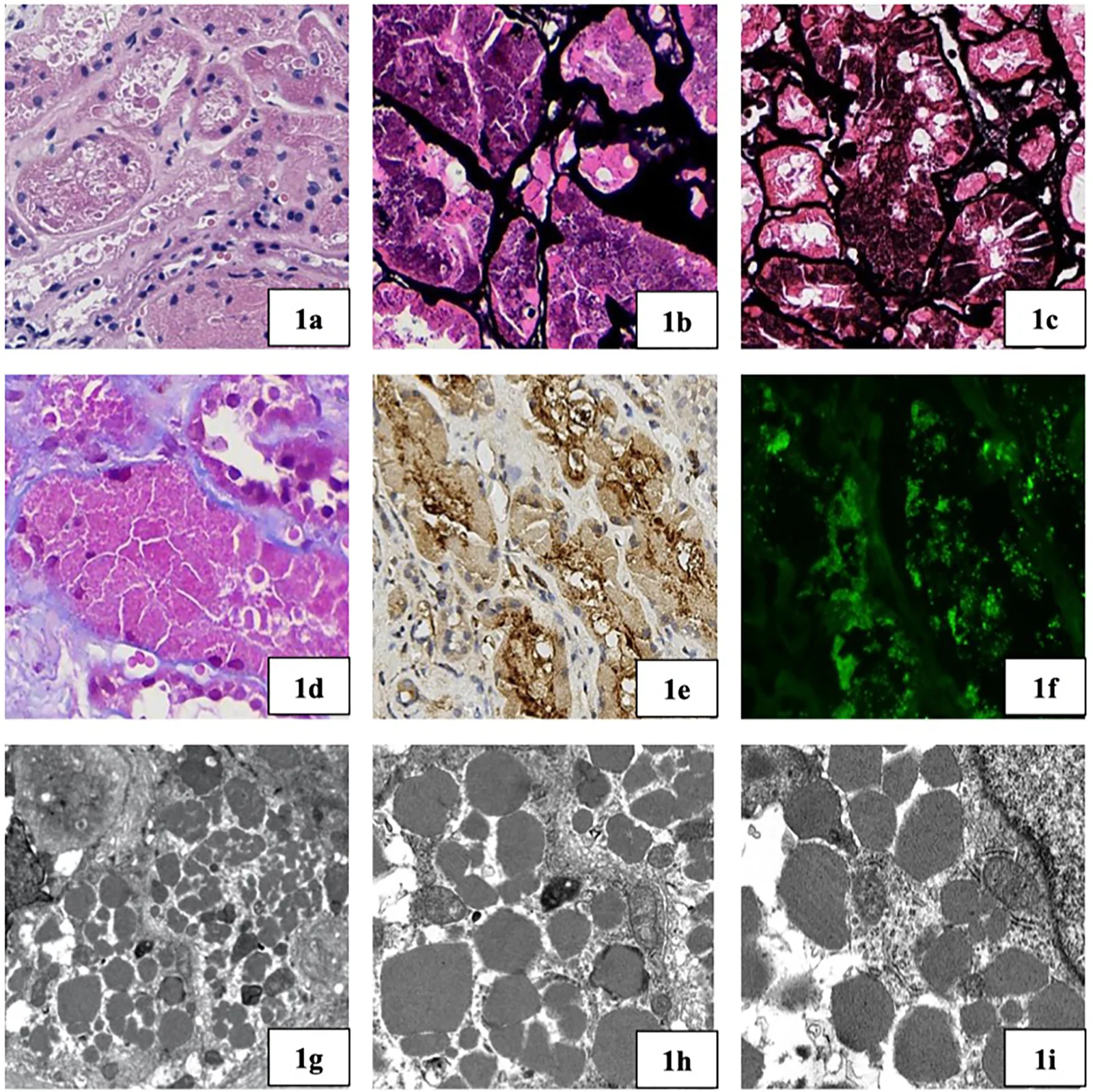
Pathological findings of case 1 (crystalline LCPT). **(a)** HE staining shows granular degeneration of proximal tubular epithelial cells. (**b-c**) PASM staining: diffuse dark brown particles in the cytoplasm. **(d)** Masson's trichrome staining: red cytoplasmic granules. **(e)** Immunohistochemistry: strong *κ* positivity. **(f)** Immunofluorescence (pronase-digested paraffin): *κ* restriction. (**g-i**) Immunoelectron microscopy: colloidal gold particles (anti-*κ*) attached to rhomboid and polygonal crystals.

Assessment of Proximal Tubular Function: The patient exhibited glycosuria (urine glucose ++), elevated urinary *α*1-microglobulin (110.9 mg/L) and *β*2-microglobulin (30.7 mg/L). Twenty-four-hour urinary phosphate, potassium, and calcium were 20.3 mmol, 39.2 mmol/24 h, and 0.53 mmol/24 h, respectively (Table 1). These findings were consistent with proximal tubular dysfunction. Urinary pH was 7.5; however, urinary amino acid analysis was not performed, and a formal diagnosis of Fanconi syndrome was therefore not established.

Diagnosis: Crystalline LCPT (IgG-*κ* type).

Treatment and Follow-up: The treatment details and adverse events are summarized in Table 2. The patient began chemotherapy on March 12, 2019, with a PAD regimen (bortezomib 2.4 mg on days 1, 4, 8, 11; liposomal doxorubicin 20 mg on days 1, 8, 11; dexamethasone 20 mg on days 1–2, 4–5, 8–9, 11–12). After completing four cycles of PAD, he underwent autologous hematopoietic stem cell transplantation (ASCT). Post-transplantation, he received two cycles of VDD (bortezomib plus liposomal doxorubicin and dexamethasone) and was then switched to an IRD regimen (ixazomib 4 mg on days 1, 8, 15; lenalidomide 25 mg on days 1–21; dexamethasone 20 mg on days 1–2, 8–9, 15–16, 22–23) starting in June 2020. He continued IRD-based maintenance and, from November 2024 to April 2026, received lenalidomide maintenance therapy.

**Table 2:** Treatment details and adverse events.

Case	Treatment line	Regimen	Dose and schedule	Cycles	Response	Adverse events
Case 1	First-line	PAD	Bortezomib 2.4 mg d1,4,8,11; liposomal doxorubicin 20 mg d1,8,11; dexamethasone 20 mg d1–2,4–5,8–9,11–12	4	CR	Acute bronchitis (resolved); no severe neuropathy or cardiotoxicity
Case 1	Consolidation	ASCT	High-dose melphalan 200 mg/m^2^	1	CR	Not systematically recorded
Case 1	Post-ASCT	VDD	Bortezomib + liposomal doxorubicin + dexamethasone	2	CR	Not systematically recorded
Case 1	Maintenance	IRD	Ixazomib 4 mg d1,8,15; lenalidomide 25 mg d1–21; dexamethasone 20 mg d1–2,8–9,15–16,22–23	Multiple	Sustained CR	Not systematically recorded
Case 1	Maintenance	Lenalidomide	Lenalidomide 25 mg d1–21 qod	2024-11 to 2026-04	Sustained CR	Not systematically recorded
Case 2	First-line	PCD	Bortezomib 2.3 mg d1,5,8,12; cyclophosphamide 0.2 g d1,5,8,12; dexamethasone 20 mg d1,5,8,12	1	Stable disease	Not systematically recorded
Case 2	Subsequent	Multiple lines	Bortezomib, cyclophosphamide, dexamethasone, ixazomib, pomalidomide, lenalidomide	Not recorded	Progressive disease	Not systematically recorded
Case 2	ASCT	Not performed	—	—	—	—

ASCT, autologous hematopoietic stem cell transplantation; CR, complete response; IRD, ixazomib–lenalidomide–dexamethasone; PAD, bortezomib–liposomal doxorubicin–dexamethasone; PCD, bortezomib–cyclophosphamide–dexamethasone; qod, every other day; VDD, bortezomib–liposomal doxorubicin–dexamethasone.

Serial monitoring showed that eGFR improved from 61.6 mL/min/1.73 m^2^ at baseline to 84.8 mL/min/1.73 m^2^ in March 2021, and remained stable at 53.9 mL/min/1.73 m^2^ at the last follow-up in April 2026. At 84 months of follow-up, serum creatinine was 113 μmol/L, with no detectable proteinuria. Both free *κ* and *λ* light chains were negative. The patient remains dialysis-independent with stable renal function.

Adverse events were not systematically recorded; the patient experienced one episode of acute bronchitis during induction, which resolved with antibiotics. No severe peripheral neuropathy or cardiac toxicity was documented.

### Case 2: Non-crystalline LCPT with concurrent light-chain cast nephropathy (IgD-*λ* restricted type)

A 64-year-old male was admitted on August 15, 2019, with nausea, vomiting, and rapidly rising serum creatinine. Laboratory results are summarized in Table 1. Relevant findings included hemoglobin 104 g/L, platelet count 130 × 10⁹/L, serum creatinine 1,021 μmol/L, eGFR 4.1 mL/min/1.73 m^2^, 24-hour urinary protein 2060mg, serum calcium 2.47 mmol/L, phosphate 1.73 mmol/L, potassium 3.8 mmol/L, bicarbonate 18.7 mmol/L, and uric acid 811 μmol/L. Serum LDH was 297 U/L at admission and peaked at 1,961 U/L on August 30, 2019. Serum free *λ* light chains were markedly elevated (31,550 mg/L), with a *κ*/*λ* ratio of 0.0003. Immunofixation electrophoresis detected a monoclonal IgD-*λ* peak; serum IgG, IgA, and IgM were all decreased (4.14, 0.24, and 0.18 g/L, respectively). MRI revealed multifocal abnormal bone marrow signal intensities. Bone marrow smear showed 77% plasma cells, and flow cytometry identified 36.7% monoclonal plasma cells with *λ* light-chain restriction. FISH analysis revealed 1q21 amplification; TP53 status was not available. The karyotype was 46, XY (20). The patient was diagnosed with IgD-*λ* multiple myeloma, DS stage III, ISS stage III, R-ISS stage III.

Renal Biopsy Findings: Hematoxylin and eosin (HE) staining showed 14 glomeruli, of which 2 were globally sclerosed. Glomerular changes were otherwise mild. Acidophilic, round inclusion bodies were observed within proximal tubular epithelial cells (Figure 2a), and these round to oval deposits stained positively with PASM (Figure 2b). Masson's trichrome staining revealed heterogeneous brick-red globular structures and laminated intracytoplasmic inclusions (Figures 2c,d). In addition, intraluminal casts were identified in some tubules, consistent with concurrent light-chain cast nephropathy. Congo red staining was negative. Immunohistochemistry showed positive staining for *λ* light chains in proximal tubule cells (Figure 2e), and immunofluorescence on protease-digested paraffin sections confirmed *λ* light-chain restriction (Figure 2f). Electron microscopy disclosed swollen proximal tubular epithelial cells containing abundant round lysosomes of varying sizes (Figures 2g–i); no crystalline structures were observed. Immunoelectron microscopy demonstrated dense colloidal gold labeling for *λ* light chains over these lysosomes, thereby confirming monoclonal *λ* light-chain deposition.

**Figure 2:**
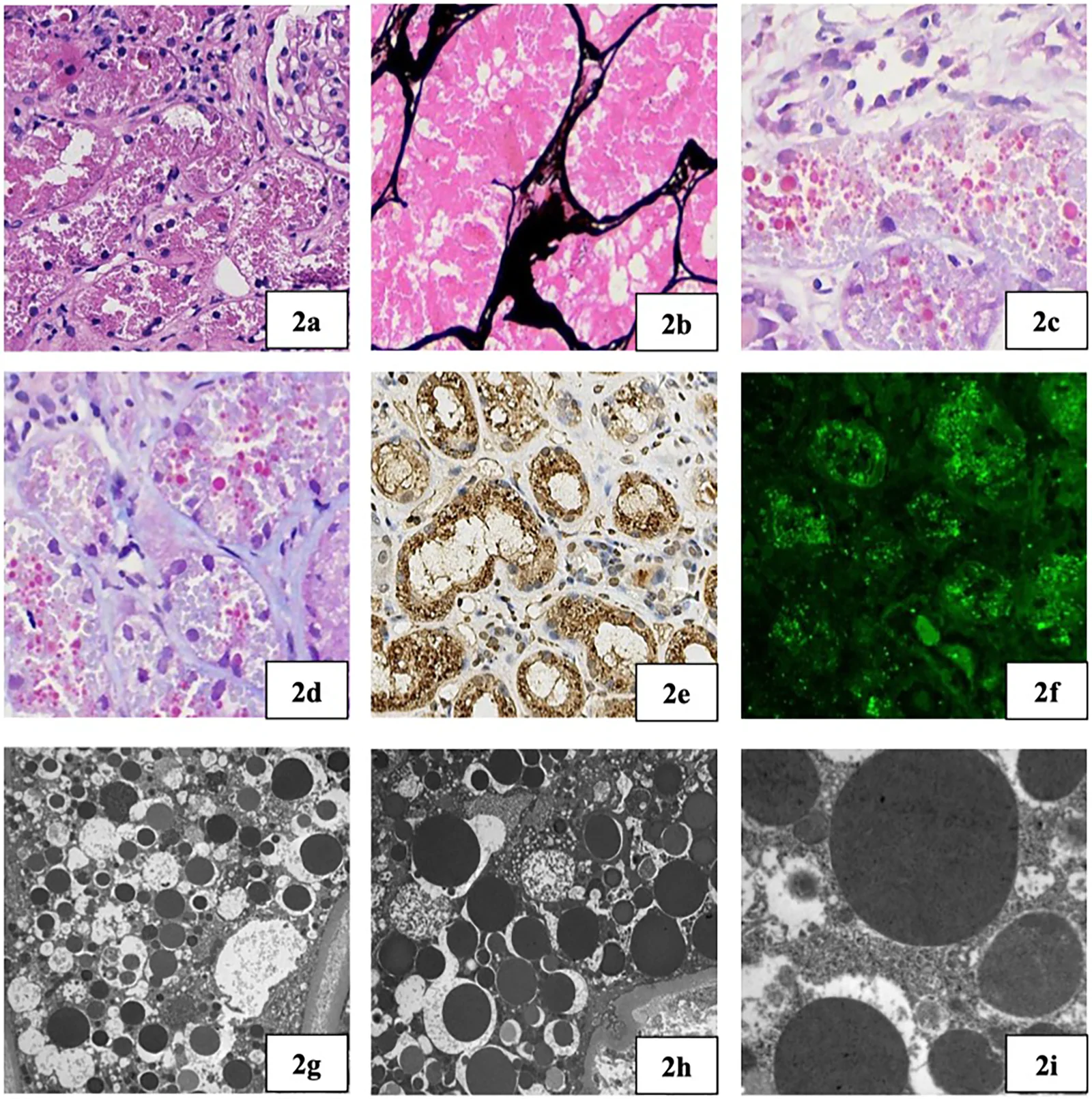
Pathological findings of case 2 (non-crystalline LCPT). **(a)** HE stains: eosinophilic round inclusions. **(b)** PASM staining: round to ovoid deposits. (**c-d**) Masson's trichrome staining: brick-red globules in the cytoplasm. **(e)** Immunohistochemistry: *λ* positivity. **(f)** Immunofluorescence (pronase-digested paraffin): *λ* restriction. (**g-i**) Immunoelectron microscopy: gold particles (anti-*λ*) decorating lysosomes; no crystals seen.

Assessment of Proximal Tubular Function: Urinary pH was 7.5, and urine glucose was negative. Serum phosphate was 1.73 mmol/L, and serum bicarbonate was 18.7 mmol/L. Urinary *β*2-microglobulin was 10.2 mg/L (Table 1). A formal diagnosis of Fanconi syndrome was not established, likely because the severe acute kidney injury (eGFR 4.1 mL/min/1.73 m^2^) and concurrent cast nephropathy obscured the classic features of proximal tubular dysfunction. Urinary amino acid analysis was not performed.

Diagnosis: Non-crystalline LCPT with concurrent light-chain cast nephropathy associated with IgD-*λ* multiple myeloma.

Treatment and Follow-up: The treatment details and adverse events are summarized in Table 2. Due to severe renal failure (eGFR 4.1 mL/min/1.73 m^2^), hemodialysis was initiated on August 16, 2019. The patient was transferred to the hematology department on August 24 and started chemotherapy while continuing hemodialysis. He remained on maintenance hemodialysis (three sessions per week) from August 16, 2019.The patient received a PCD regimen (bortezomib 2.3 mg on days 1, 5, 8, 12; cyclophosphamide 0.2 g on days 1, 5, 8, 12; dexamethasone 20 mg on days 1, 5, 8, 12) as first-line therapy, starting August 27, 2019. He subsequently received multiple lines of chemotherapy, including bortezomib, cyclophosphamide, dexamethasone, ixazomib, pomalidomide, and lenalidomide. However, due to rapid disease progression and poor performance status, ASCT was not performed. In June 2022, he was transitioned to palliative care, and laboratory monitoring was discontinued. He continued hemodialysis until July 15, 2022, when dialysis was discontinued because of recurrent convulsions. He died on July 17, 2022, from multiple organ failure, after a total follow-up of approximately 35 months. Adverse events were not systematically recorded.

## Discussion

The two LCPT cases reported herein illustrate the morphological and clinical divergence between crystalline and non-crystalline subtypes. However, these cases also differ in several important confounding factors, including light-chain burden (427.5 vs. 31,550 mg/L), initial eGFR (61.6 vs. 4.1 mL/min/1.73 m^2^), presence of concurrent cast nephropathy, tumor stage, and treatment intensity. Therefore, the divergent outcomes cannot be attributed solely to crystalline versus non-crystalline morphology. Rather, these cases highlight the diagnostic and therapeutic challenges of LCPT and the need for individualized management.

In the related entity of light-chain crystalline podocytopathy (LCCP), serum monoclonal immunoglobulin was found to be IgG-*κ* in 86% of cases in one series (5). Similarly, crystalline LCPT is predominantly associated with *κ* light-chain restriction, particularly the V*κ*1 subtype, whose hydrophobic side chains confer resistance to lysosomal degradation and promote crystal formation (9). Consequently, the majority of reported crystalline LCPT cases are of the IgG-*κ* isotype (9).

In contrast, cases attributed to *λ* light chains are rarely reported (8, 10). A notable example is non-crystalline LCPT in IgD-*λ* multiple myeloma, a rare and aggressive subtype (8, 10). Because *λ* light chains lack hydrophobic side chains that protect against lysosomal degradation, they are excessively internalized via receptor-mediated endocytosis, resulting in lysosomal overload and non-crystalline proximal tubular injury.

Consequently, this process leads to lysosomal dilatation and promotes non-crystalline proximal tubular injury. In this regard, Larsen et al. reviewed 13 LCPT cases, of which 10 were non-crystalline, and 9 of the 10 showed *λ* light-chain restriction (11). These observations underscore that the morphological patterns of light chain–induced proximal tubular lesions are closely linked to the specific monoclonal light-chain subtype. In addition, renal tubular damage, although rare, has also been reported in patients with Waldenström macroglobulinemia (12, 13).

In patients with plasma cell neoplasms or MGRS detected on renal biopsy, careful interpretation of *κ* and *λ* immunofluorescence positivity is essential. LCPT should be considered a likely cause of renal dysfunction. Based on the clinical courses of the present two cases, crystalline LCPT appears to be associated with relatively mild tubular injury and slower progression. Early intervention, particularly by means of stem cell transplantation, can effectively suppress tumor proliferation and ameliorate renal impairment.

In Case 2, concurrent light-chain cast nephropathy was identified in addition to non-crystalline LCPT. The presence of cast nephropathy, together with a very high *λ* light-chain burden, likely contributed significantly to the severe and irreversible renal failure requiring maintenance hemodialysis. This confounding factor should be considered when interpreting the renal outcome.

Immunoglobulin D multiple myeloma is a rare subtype, comprising less than 2% of all multiple myeloma cases (6). It is characterized by a younger age at onset compared with other subtypes (6) and carries a significantly higher risk of renal failure, which is the most common complication and a major contributor to its poor prognosis. Yan et al. reported that among 85 newly diagnosed IgD MM patients, renal insufficiency was present in 55.3%, with 59.6% presenting with acute renal failure as the initial manifestation (14). Patients with renal insufficiency had significantly shorter median overall survival than those without (29 months vs. > 40 months, *p* = 0.042), and renal insufficiency was identified as an independent adverse prognostic factor (HR = 6.14, 95% CI: 1.39–27.13, *p* = 0.016) (14).

In IgD-type multiple myeloma, the light-chain type is predominantly *λ*-restricted, with reported frequencies of 65% in one series (6) and up to 90% in another (15). As discussed by Lan et al., studies have shown that *κ* light chains of the V*κ*1 subgroup are associated with intracellular crystal formation, whereas certain *λ* light chains typically do not form crystals (4). There is a strong association between non-crystalline tubular injury and severe renal failure in IgD-*λ* multiple myeloma, with rapid progression and inferior treatment outcomes compared with crystalline *κ*-type disease. Consequently, the distinct morphological features observed on renal biopsy—crystalline versus non-crystalline—carry important clinical and prognostic implications.

In recent years, therapeutic strategies for LCPT have progressed considerably. The overarching principle is to suppress abnormal light-chain production as early as possible while providing supportive care to preserve renal function. Bortezomib-based targeted chemotherapy regimens are now widely used. Reece et al. reported that among 36 IgD myeloma patients who underwent autologous hematopoietic cell transplantation (HCT), the 3-year progression-free and overall survival probabilities were 38% and 69%, respectively, comparable to those of IgG and IgA subtypes (16). Furthermore, Li et al. conducted a systematic review and meta-analysis of 26 studies, finding that with reduced-dose melphalan (≤140 mg/m^2^), ASCT is safe and effective for myeloma patients with renal insufficiency, yielding comparable complete response rates and progression-free survival to those with normal renal function, although overall survival remained significantly poorer (17). The International Myeloma Working Group (IMWG) consensus statement recommends that high-dose melphalan (140 mg/m^2^) and ASCT be considered for younger patients with chemosensitive disease (18). However, in a large European Society for Blood and Marrow Transplantation (EBMT) registry study of 2,802 patients undergoing ASCT, IgD myeloma was identified as an independent prognostic marker associated with the worst progression-free and overall survival among all myeloma subtypes (19).

In the present study, the treatment strategy for Case 1 was determined by his younger age, preserved performance status, and chemosensitive disease, which made him eligible for PAD induction followed by ASCT (Figure 3a). In contrast, Case 2 presented with severe acute kidney injury requiring hemodialysis and a high tumor burden; therefore, a PCD regimen was selected, and ASCT was not performed because of rapid disease progression and poor performance status (Figure 3b). These differences highlight the need for individualized treatment in LCPT. Regarding adverse events, the main class-specific toxicities of the agents used include peripheral neuropathy with bortezomib, cardiotoxicity with liposomal doxorubicin, and hematologic toxicity with lenalidomide and ixazomib. In Case 1, no severe peripheral neuropathy or cardiotoxicity was documented; in Case 2, adverse events were not systematically recorded. Detailed treatment-related toxicities are summarized in Table 2. Mass spectrometry was not performed due to limited biopsy tissue availability, and this is acknowledged as a limitation.

**Figure 3:**
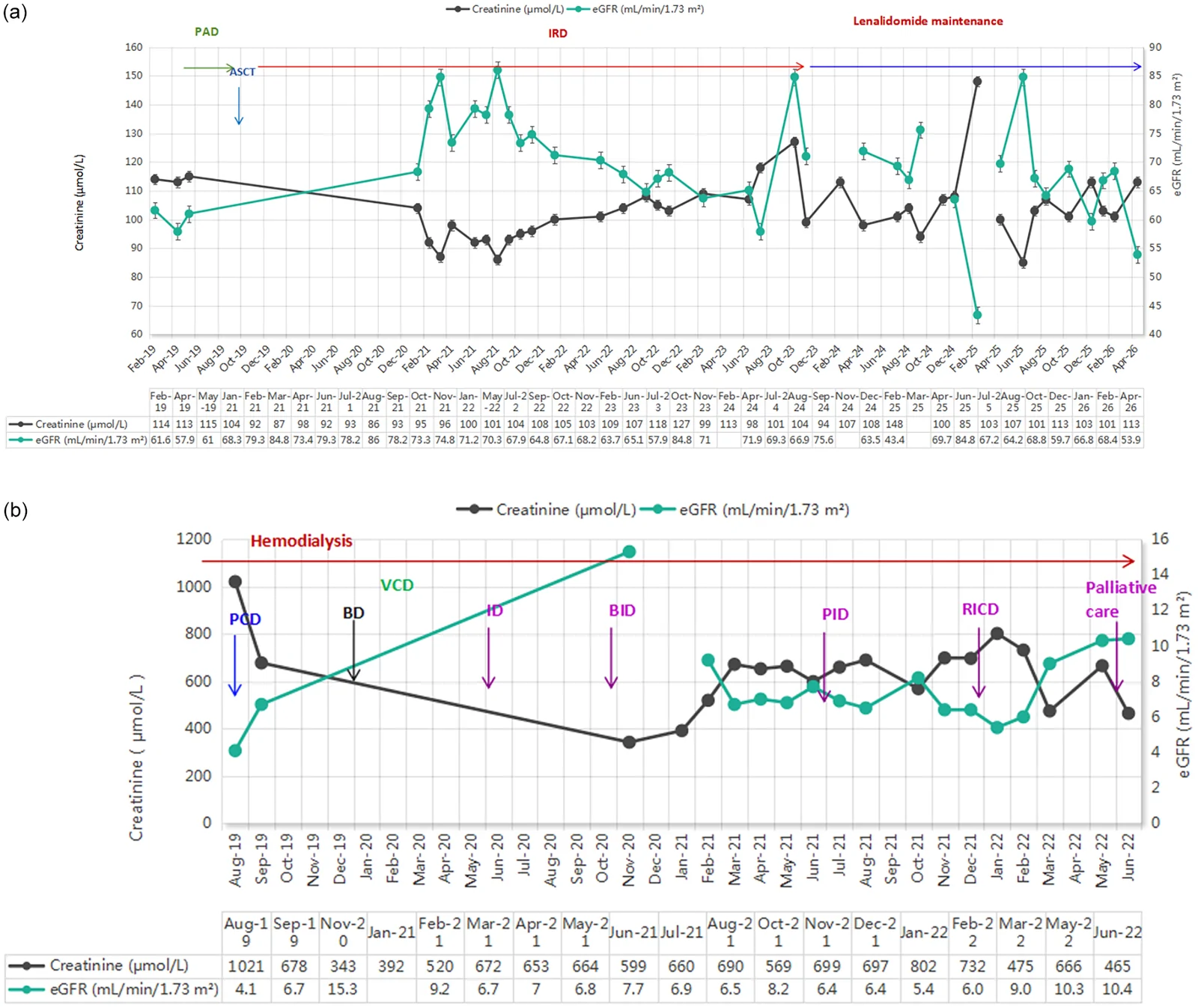
Timeline of interventions and renal function in the two cases. (**a**) Case 1: Serial serum creatinine (solid line, left y-axis) and eGFR (dashed line, right y-axis) from 2019 to 2026. Arrows indicate the initiation of PAD chemotherapy (March-June 2019), autologous hematopoietic stem cell transplantation (ASCT; November 2019), IRD therapy (June 2020–October 2024), and lenalidomide maintenance (November 2024–April 2026). (**b**) Case 2: Serial serum creatinine (solid line, left y-axis) and eGFR (dashed line, right y-axis) from 2019 to 2022. Arrows indicate admission (August 15, 2019), initiation of hemodialysis (August 16, 2019), and the sequential administration of chemotherapy regimens including PCD (August 27, 2019), VCD, BD, ID, BID, PID, and RICD. The last available laboratory data point was in June 2022, after which the patient was transitioned to palliative care. The patient remained on hemodialysis until July 15, 2022, when dialysis was discontinued, and he died on July 17, 2022.

In Case 1, the patient achieved complete hematologic remission at 84 months following ASCT combined with bortezomib-based induction and maintenance therapy, with stable renal function. In contrast, Case 2 did not receive ASCT; despite multiple chemotherapy regimens, renal function continued to deteriorate, ultimately leading to death. In patients with significant renal impairment, the decision to proceed with ASCT warrants careful evaluation. In such patients, melphalan dose reduction to 140 mg/m^2^ is recommended, and ASCT has been shown to be safe and effective with acceptable toxicity (17). Nevertheless, IgD myeloma remains the subtype with the poorest post-ASCT prognosis (19). In patients with light chain–associated Fanconi syndrome, renal function remained stable as long as hematologic response was maintained and declined when serum free light chain levels rebounded, emphasizing the importance of sustained hematologic control for renal preservation (20). Hence, when a patient is diagnosed with IgD-type multiple myeloma accompanied by renal impairment, and provided the patient's general condition is stable, early referral for ASCT should be strongly considered.

Limitations: This study has several limitations. Only two cases are presented, and the small number of cases precludes statistical comparison or causal inference. The two cases differed in light-chain burden, initial renal function, presence of cast nephropathy, tumor stage, and treatment. Proximal tubular functional parameters were not uniformly available, and urinary amino acid analysis was not performed. Mass spectrometry was not performed in this retrospective study. Adverse events were not systematically recorded in Case 2. The association between light-chain isotype and morphological phenotype is largely based on published cohorts and should be interpreted with caution.

Despite these limitations, this report provides detailed morphological and clinical characterization of two rare LCPT subtypes and underscores the importance of individualized management in IgD-*λ* non-crystalline LCPT with concurrent cast nephropathy.

## Summary

This report delineates the renal pathological features of two LCPT cases, with and without crystal formation. In patients presenting with unexplained proximal renal tubular dysfunction and concurrent monoclonal gammopathy, a high index of suspicion for LCPT should be entertained. Crystalline LCPT is commonly associated with IgG-*κ* light-chain deposition, whereas non-crystalline LCPT is closely linked to IgD-*λ* light-chain deposition. Early diagnosis, based on electron microscopy and trypsin-digested paraffin immunofluorescence, is critical.

Non-crystalline LCPT in the setting of IgD-*λ* requires particular vigilance, because it is associated with more rapid progression of tubular injury. The primary treatment for LCPT consists of a bortezomib-based regimen combined with autologous hematopoietic stem cell transplantation.

## Data Availability

The raw data supporting the conclusions of this article will be made available by the authors, without undue reservation.
