# Supplementary material for: Crystalline versus non-crystalline light-chain proximal tubulopathy: a case series
Source: Front Med (Lausanne). 2026 Oct 14;13:1963349. doi: 10.3389/fmed.2026.1963349 (PMC13713427; doi:10.3389/fmed.2026.1963349)
Supplement: Supplementary file 1 [file Supplementaryfile1.docx]

**Supplementary Materials**

**Crystalline versus Non-Crystalline Light-Chain Proximal Tubulopathy**

**Authors**

Zhi-Rong Zhang^1†^, Li-Mei Wang^1†^, Xiao-Le Meng^2†^*, Ting Zhu^1†^, Zhi-Gang Zhang^3^, Zhong-Hua Zhao^3^, Wei-Xia Wang^1^*, Si-Nian Huang^1^*

**Affiliations**

^1^Department of Pathology, QingPu Branch, Zhongshan Hospital Affiliated to Fudan University, Shanghai, 201700, China

^2^Department of Pathology, Zhongshan Hospital (Xiamen Branch), Fudan University; Clinical Research Center for Precision Medicine of Abdominal Tumor of Fujian Province, Xiamen, 361015, China

^3^Department of Pathology, School of Basic Medical Sciences, Fudan University, Shanghai, 200032, China

^†^These authors have contributed equally to this work.

**Running head:** **Crystalline vs. Non-Crystalline LCPT**

**Table S1.** Proximal tubular function parameters

| Parameter | Case 1 | Case 2 |
| --- | --- | --- |
| Urinary pH | 7.5 | 7.5 |
| Urinary α1-microglobulin (mg/L) | 110.9 | Not performed |
| Urinary β2-microglobulin (mg/L) | 30.7 | 10.2 |
| 24-h urinary phosphate (mmol) | 20.3 | 6.5 |
| 24-h urinary potassium (mmol) | 39.2 | 11.4 |
| 24-h urinary calcium (mmol) | 0.53 | 0.29 |
| Urinary amino acids | Not performed | Not performed |

**Table S2.** Comparison of serial serum LDH levels between the two cases
